# Cutaneous thermosensitivity differences among the face, hand or thigh appear not to exist for skin blood flow during normothermic states

**DOI:** 10.1186/2046-7648-4-S1-A116

**Published:** 2015-09-14

**Authors:** Catriona A Burdon, Kyoko Tagami, Joonhee Park, Joanne N Caldwell, Nigel AS Taylor

**Affiliations:** 1 Centre for Human and Applied Physiology, School of Medicine, University of Wollongong, Wollongong, Australia

## Introduction

Variations in the volume of the sensory cortex (homunculus) assigned to different skin regions may lead one to postulate that thermal stimulation of some body segments (*e.g*. face, hand) may evoke more powerful autonomic responses. That is, there may exist a mosaic of cutaneous thermosensitivity. To study thermosensitivity, thermal feedback from sites other than the treated site needs to be minimised. This is achieved via whole-body clamping of deep-body and skin temperatures. Previously, greater sensitivity of the face was observed for sudomotor control in mildly hyperthermic individuals [1], so the aim of this investigation was to explore possible sensitivity variations in the control of skin blood flow during isolated stimulation of three sites, but after a normothermic clamp had been established.

## Methods

Nine subjects (5 males, 4 females) participated in two trials with limb-segment blood flow measured at the hand and forearm, but in separate trials (right side; water-displacement plethysmography). Deep-body (aural) and skin temperatures were clamped at normothermic levels using a whole-body, water-perfusion suit. The three treated skin sites (face, left hand, left thigh) were then stimulated using individual water-perfusion patches of the same surface area, to both elevate and reduce local skin temperature ~5°C from baseline temperatures.

## Results

Mean body temperature (36.7 °C, SD 0.2) for the two trials was not different (*P *> 0.05), verifying successful clamping. Local heating (4.9 °C, SD 1.1) increased, while cooling (-5.2 °C, SD 1.2) depressed both hand and forearm blood flows (*P *< 0.05 for all comparisons). There were no differences in the size of these responses to either thermal treatment, regardless of the skin site stimulated. Furthermore, whilst hand and forearm blood flows were not identical, their responses were of equivalent magnitude (*P *> 0.05). Therefore, these changes were combined, with the resulting data contained in Table 1.

**Table 1:** Change in hand and forearm blood flow during thermal stimulation of the face, hand and thigh.

Treated site	Hand and forearm blood flow changes (mL.100 mL tissue^-1^.min^-1^)	
	**Heating**	**Cooling**

Face	2.73 (SD 1.65)	-1.54 (SD 1.49)

Hand	3.40 (SD 2.98)	-1.59 (SD 1.00)

Thigh	1.93 (SD 1.81)	-2.17 (SD 2.02)

## Discussion

As expected, thermal stimulation produced significant effector responses. However, as opposed to previous observations, this did not differ among sites when subjects were normothermic. It is therefore concluded that local differences in cutaneous thermosensitivity appear not to exist with respect to skin blood flow modulation in this thermal state, at least for those sites investigated. In an accompanying communication, this question is addressed again, but now with subjects in a mildly hyperthermic state.
